# Unraveling ChR2-driven stochastic Ca^2+^ dynamics in astrocytes: A call for new interventional paradigms

**DOI:** 10.1371/journal.pcbi.1008648

**Published:** 2021-02-10

**Authors:** Arash Moshkforoush, Lakshmini Balachandar, Carolina Moncion, Karla A. Montejo, Jorge Riera

**Affiliations:** 1 Department of Biomedical Engineering, Florida International University, Miami, Florida, United States of America; 2 Department of Neurologic Surgery, Mayo Clinic, Rochester, Minnesota, United States of America; Inria, FRANCE

## Abstract

Optogenetic targeting of astrocytes provides a robust experimental model to differentially induce Ca^2+^ signals in astrocytes *in vivo*. However, a systematic study quantifying the response of optogenetically modified astrocytes to light is yet to be performed. Here, we propose a novel stochastic model of Ca^2+^ dynamics in astrocytes that incorporates a light sensitive component—channelrhodopsin 2 (ChR2). Utilizing this model, we investigated the effect of different light stimulation paradigms on cells expressing select variants of ChR2 (wild type, ChETA, and ChRET/TC). Results predict that depending on paradigm specification, astrocytes might undergo drastic changes in their basal Ca^2+^ level and spiking probability. Furthermore, we performed a global sensitivity analysis to assess the effect of variation in parameters pertinent to the shape of the ChR2 photocurrent on astrocytic Ca^2+^ dynamics. Results suggest that directing variants towards the first open state of the ChR2 photocycle (o_1_) enhances spiking activity in astrocytes during optical stimulation. Evaluation of the effect of Ca^2+^ buffering and coupling coefficient in a network of ChR2-expressing astrocytes demonstrated basal level elevations in the stimulated region and propagation of calcium activity to unstimulated cells. Buffering reduced the diffusion range of Ca^2+^ within the network, thereby limiting propagation and influencing the activity of astrocytes. Collectively, the framework presented in this study provides valuable information for the selection of light stimulation paradigms that elicit desired astrocytic activity using existing ChR2 constructs, as well as aids in the engineering of future application-oriented optogenetic variants.

## Author summary

Optogenetics is a novel technique involving the targeted delivery of light-sensitive ion channels like Channelrhodopsin-2 (ChR2) to cells. Recently, this technique has been expanded to non-neuronal cell types, e.g., astrocytes. Optogenetic control of astrocytes *in vivo* can aid in further understanding the intricacies of their debated roles in the brain. Here, using a mathematical model, we evaluate the effect of short and long-term light stimulation of ChR2-expressing astrocytes on their Ca^2+^ spiking activity and basal level. We further investigate how ChR2 gating dynamics, buffering, and coupling coefficient of Ca^2+^ influence astrocytic activity in a single cell and a network. Results of our study can serve as a tool for experimental design and engineering of new application-oriented optogenetic constructs targeting astrocytes.

## Introduction

The role of astrocytic calcium signaling in various regulatory mechanisms in the brain is far from being fully understood and is a subject of considerable controversy [see refs [1–6]]. These are fueled in part by the limited *in vivo* reproducibility of *in vitro*/*in situ* experimental observations [7–10], as well as a dearth of cell-specific protocols to induce astrocytic Ca^2+^ signaling *in vivo* in order to delineate their role from ongoing neuronal activity. Sensory and transcranial direct current stimulation techniques [11–13] have been used to elicit Ca^2+^ changes in astrocytes. However, these methods lack cell specificity due to the concurrent activation of other cell types, including neurons. Studies have also modulated astrocytic Ca^2+^ activity *in vivo* using cell-specific techniques including Ca^2+^ uncaging [14,15], chemogenetics [16,17] and optogenetics [18,19]. Ca^2+^ uncaging requires invasive site-specific delivery of calcium vehicles, which are rendered inoperative upon depletion via photolysis. Chemogenetics, e.g., designer receptor exclusively activated by designer drugs (DREADDS), offers a platform for controlled, targeted drug delivery; however, they suffer from low temporal resolution. Contrarily, optogenetics is an avant-garde, minimally invasive, and reproducible approach [20,21], providing a platform to genetically target specific cell types with high temporal and spatial precision, which can be employed as a tool to exclusively modulate astrocytic Ca^2+^ signaling *in vivo*.

Despite the recent inception of the field of optogenetics, a wide variety of optogenetic tools have been constructed, among which channelrhodopsin 2 (ChR2) has been one of the most commonly used. Applications of this technique in astrocytes has helped examine the role of these cells in memory enhancement [22], cortical state switching [23], and hyperemic response [18,19]. The biophysical characterization and the response to light stimulation in several ChR2 variants, predominantly in excitable cells, are available in literature [24,25]. More recently, several ChR2 variants have been engineered for enhanced channel conductance (ChETA) [26], increased calcium permeability (CaTCh) [27], and faster recovery kinetics (ChRET/TC) [28]. ChR2 constructs have also been modified to form chimeric variants to regulate responses and facilitate multiwavelength optogenetics in neurons [29]. Contrary to neurons, a holistic approach to quantify the effect of light stimulation on astrocytes has not yet been formulated. Given that light activation of ChR2-enabled astrocytes alters dynamics of intracellular ionic species, mathematical modeling can be of importance in predicting how laser specifications, as well as the biophysical properties of the ChR2 construct, can affect astrocytic calcium signaling. Available theoretical models of Ca^2+^ dynamics in astrocytes primarily rely on elevating intracellular IP_3_ levels to initiate a Ca^2+^ response from the intracellular stores and have not evaluated the effect of direct influx of these ions through transmembrane channels, e.g. ChR2. To achieve predictions with high accuracy, it is also imperative that the model accounts for the stochastic nature of spontaneous calcium oscillations in these cells, which result from the random opening of the inositol trisphosphate receptor (IP_3_R) channels in clusters. Such a model can guide experimentalists in optimizing light stimulation paradigms for existing optogenetic variants to achieve desired Ca^2+^ levels in astrocytes, as well as aid in the development of novel application-based constructs targeting these cells.

To this end, we outline a novel stochastic model of astrocytic calcium dynamics with an incorporated optogenetic component—ChR2. Firstly, we quantify and evaluate the effect of different light stimulation paradigms on the Ca^2+^ dynamics of single cells expressing three existing ChR2 variants, i.e., wild type (WT), ChETA, and ChRET/TC. For the WT variant, we use the two channel characterizations provided in ref. [30], namely WT_1_ and WT_2_. Using the model, we analyze the effect of light stimulation in astrocytes congruent with experimental recording durations (in the order of tens of minutes) [31–33], and also gauge the potential effect of long-term optogenetic stimulation (in the order of several hours or beyond) in these cells. Secondly, we quantify the effect of varying stimulation light intensities on a ChR2-incorporated astrocyte with respect to its spiking rate and basal level. Thirdly, to identify key features necessary for the development of prospective ChR2 constructs, we perform a global sensitivity analysis of different parameters of the single cell model to the model output. Lastly, through the incorporation of gap junctions allowing for the diffusion of IP_3_ and Ca^2+^, we analyze the effect of local light stimulation on the global Ca^2+^ response in a network of astrocytes expressing ChR2.

## Materials and methods

**Fig 1:**
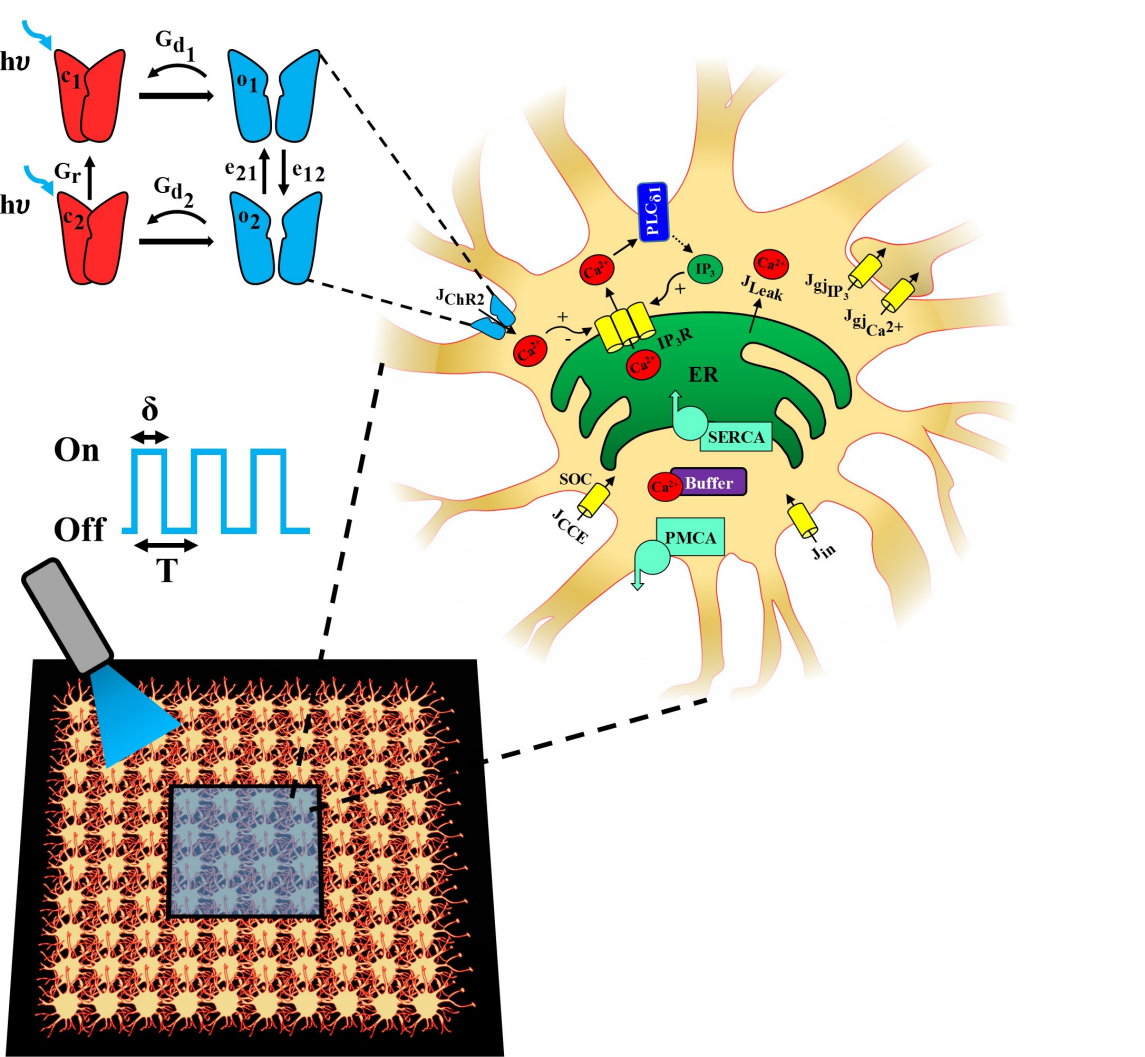
Schematic of the biophysical model. A model for a ChR2-expressing astrocyte is presented, accounting for: 1) Ca^2+^ release from the endoplasmic reticulum (ER) into the cytosol via the IP_3_R clusters, 2) Phospholipase-C δ1 (PLC_δ1_) mediated production of IP_3,_ 3) capacitive calcium entry (CCE) via the store operated calcium channel (SOC), 4) passive leak from the ER to the cytosol (J_leak_), 5) replenishment of ER stores via sarco(endo)plasmic reticulum Ca^2+^-ATPase (SERCA) pump, 6) extrusion of Ca^2+^ by plasma membrane Ca^2+^ ATPase (PMCA) pump into the extracellular (EC) space, 7) passive leak (J_in_) into the cytosol from the EC space, and 8) Ca^2+^ buffering by endogenous buffer proteins. In astrocytic network simulations (bottom panel), each cell is connected to its neighboring cells though Ca^2+^ and IP_3_ permeable gap junctions, indicated as $J_{{\mathrm{gj}}_{{\mathrm{Ca}}^{2+}}}$ and $J_{{\mathrm{gj}}_{{\mathrm{IP}}_{3}}}$, respectively, and a central region (blue shaded box) is stimulated with light. A 4-state model [closed states (c_1_ and c_2_) in red and open states (o_1_ and o_2_) in blue] is used to represent ChR2 gating dynamics. The blue light (λ = 470 nm) stimulation paradigm used to open ChR2, leading to a Ca^2+^ influx (J_ChR2_), is characterized by pulse period (T) and pulse width (δ).

The biophysical model outlined in Fig 1 consists of our previously developed model of astrocytic calcium dynamics [34,35], based on the Li-Rinzel simplification of the De Young-Keizer model [36,37], and a 4-state model of ChR2 photocurrent kinetics adapted from refs. [30,38]. We incorporated a 4-state model of ChR2 photocycle, as previous studies have demonstrated its superiority in capturing the dynamics of the ChR2 photocurrent compared to 3-state models [30,39]. The ChR2 model assumes two sets of intra-transitional closed/open states, i.e., dark-adapted (c_1_ and o_1_) and light-adapted (c_2_ and o_2_), in describing the dynamics of a ChR2 photocycle. Light stimulation window (S_0_(t)) is modeled as a pulsed train with unit amplitude and is characterized by a period (T) and a pulse width (δ) (expressed as a percentage of T), indicating the duration within the pulse period for which the light is on.

The model incorporates Ca^2+^ influx from the extracellular space through a light-evoked ChR2 flux (J_ChR2_), a capacitive calcium entry (CCE) flux via store-operated Ca^2+^ channels (SOC) (J_CCE_), and a leak flux representing other transmembrane Ca^2+^ channels and exchangers (J_in_). It also accounts for the release of Ca^2+^ from the endoplasmic reticulum (ER) into the cytosol through IP_3_Rs ($J_{{\mathrm{IP}}_{3}R}$), a form of Ca^2+^-induced Ca^2+^ release (CICR) process, and a leak flux (J_Leak_). Replenishment of ER Ca^2+^ is done via activation of the sarco(endo)plasmic reticulum Ca^2+^-ATPase (SERCA) pump, while plasma membrane Ca^2+^ ATPase (PMCA) pump extrudes cytosolic Ca^2+^ into the extracellular space. The dynamics of IP_3_ concentration in the soma ([IP_3_]) is included via Ca^2+^-dependent activation of the Phospholipase C_δ1_ (PLC_δ1_) pathway. Buffering of Ca^2+^ is explicitly accounted for by a fast buffering approximation process [40]. Intercellular signaling in a network of astrocytes is modeled by incorporation of Ca^2+^ and IP_3_ permeable gap junctions between neighboring cells.

### Single cell model

The dynamics of a single ChR2-enabled astrocyte can be summarized by the following system of stochastic differential equations (SDEs):

dx=f(t,x,p)dt+G(t,x,p)dω (1)

where **x** = ([Ca^2+^]_c_, [IP_3_], h, [C_o_], o_1_, o_2_, c_1_, c_2_, s)^T^ is the vector of state variables, [Ca^2+^]_c_ is the cytosolic calcium concentration, [C_o_] is the total Ca^2+^ concentration in the cell, i.e., cytosol and ER, h is the fraction of open inactivation gates of IP_3_R channels, and s captures the temporal kinetics of conformational changes of ChR2 following light stimulation. **p** denotes the vector of model parameters summarized in Table 1. Components of the **f** vector, i.e., the drift rate function, contain deterministic equations of the single cell model and are described in detail in later sections. **G** is a diagonal matrix of diffusion rate components of the stochastic processes (noise) associated with state variables. The Brownian motion vector is denoted as d**ω**.

**Table 1:** Model parameters.

	Value		Unit	Description	Source
**IP**_**3**_ **Dynamics**					
**v**_**δ**_	0.15		μM/s	Maximum rate of IP_3_ production (PLC_δ1_)	[35]
**K**_**δCa**_	0.55		μM	Half saturation constant of Ca^2+^ (PLC_δ1_)	[35]
$K_{{\mathrm{IP}}_{3}}$	1.25		s^-1^	IP_3_ degradation rate	[35]
$X_{{\mathrm{IP}}_{3}}$	0.14		μM/s	Basal level of cytosolic IP_3_ production	[35]
**Ca**^**2+**^ **Dynamics**					
**x**_**CCE**_	0.01		μM/s	Maximum CCE influx	[35]
**h**_**CCE**_	10		μM	Half-inactivation constant for CCE influx	[35]
**α**_**1**_	0.19		~	Volume ratio between ER and cytosol	[35]
**v**_**SERCA**_	0.90		μM/s	Maximum rate constant of SERCA pump	[35]
**K**_**p**_	0.10		μM	Half-maximal activation of the SERCA pump	[35]
**d**_**1**_	0.13		μM	Dissociation constant for IP_3_ (IP_3_R)	[35]
**d**_**5**_	0.08		μM	Ca^2+^ activation constant (IP_3_R)	[35]
**v**_**1**_	6		s^-1^	Ligand-operated IP_3_R channel flux constant	[35]
**v**_**2**_	0.11		s^-1^	Ca^2+^ passive leakage flux constant	[35]
**k**_**out**_	0.50		s^-1^	Rate constant of Ca^2+^ extrusion	[35]
**λ**	1		~	Time scaling factor	[35]
**ε**	0.01		~	Ratio of PM to ER membrane surface area	[35]
**J**_**in**_	0.04		μM/s	Passive leakage	[35]
**V**_**m**_	-70		mV	Membrane voltage	Model assumption
**G(V**_**m**_**)**	$\frac{\left(10.6–14.6{e\mathrm{xp}}^{-(\frac{V_{m}}{42.7})}\right)}{V_{m}}$		~	Voltage dependent rectification function	[38]
**vol**_**cyt**_	10^-12^		L	Volume of the cytosol, assuming spherical cell	[41]
**A**_**m**_	4.83×10^-6^		cm^2^	Surface area of the astrocyte membrane (calculated using *vol*_*cyt*_ and assuming spherical cell shape)	Model assumption
**F**	9.65×10^4^		C/mol	Faraday’s constant	
$z_{{\mathrm{Ca}}^{2+}}$	2		~	Valence of Ca^2+^ ions	
**b**_**t**_	200		μM	Total buffer protein concentration	[42]
**K**	20		μM	Buffer rate constant ratio	[42]
**N**	20		~	Number of IP_3_Rs within a cluster	[43]
**Gating Parameters**					
**a**	0.20		(μMs)^-1^	Rate constant for Ca^2+^ binding in IP_3_ inhibitory site	[35]
**d**_**2**_	1.05		μM	Dissociation constant for Ca^2+^ inhibition (IP_3_R)	[35]
**d**_**3**_	0.94		μM	Dissociation constant for IP_3_ (IP_3_R)	[35]
**Network Dynamics**					
$D_{{\mathrm{IP}}_{3}}$	1		s^-1^	Coupling coefficient of IP_3_	Model estimate
$D_{{\mathrm{Ca}}^{2+}}$	0.1		s^-1^	Coupling coefficient of Ca^2+^	Model estimate
**Wiener Processes**					
$\sigma_{{\mathrm{IP}}_{3}}$	0.02		s^-1/2^	Variance of Wiener process of [IP_3_]	[35]
$\sigma_{{\mathrm{Ca}}^{2+}}$	0.01		s^-1/2^	Variance of Wiener process of [Ca^2+^]_c_	[35]
$\sigma_{c_{o}}$	0.01		s^-1/2^	Variance of Wiener process of [C_o_]	[35]
**ChR2 Parameters**					
**p**_**1**_	**ChRWT**_**1**_	0.06	ms^-1^	Maximum excitation rate of c_1_	[26,28,30]
	**ChRWT**_**2**_	0.12			
	**ChETA**	0.07			
	**ChRET/TC**	0.13			
$G_{d_{1}}$	**ChRWT**_**1**_	0.46	ms^-1^	Rate constant for the o_1_ to c_1_ transition	[26,28,30]
	**ChRWT**_**2**_	0.01			
	**ChETA**	0.01			
	**ChRET/TC**	0.01			
**e**_**12**_	**ChRWT**_**1**_	0.20	ms^-1^	Rate constant for the o_1_ to o_2_ transition	[26,28,30]
	**ChRWT**_**2**_	4.38			
	**ChETA**	10.51			
	**ChRET/TC**	16.11			
**e**_**21**_	**ChRWT**_**1**_	0.01	ms^-1^	Rate constant for the o_2_ to o_1_ transition	[26,28,30]
	**ChRWT**_**2**_	1.60			
	**ChETA**	0.01			
	**ChRET/TC**	1.09			
**p**_**2**_	**ChRWT**_**1**_	0.06	ms^-1^	Maximum excitation rate of c_2_	[26,28,30]
	**ChRWT**_**2**_	0.01			
	**ChETA**	0.06			
	**ChRET/TC**	0.02			
$G_{d_{2}}$	**ChRWT**_**1**_	0.07	ms^-1^	Rate constant for the o_2_ to c_2_ transition	[26,28,30]
	**ChRWT**_**2**_	0.12			
	**ChETA**	0.15			
	**ChRET/TC**	0.13			
**G**_**r**_	**ChRWT**_**1**_	9.35×10^-5^	ms^-1^	Recovery rate of the c_1_ state after light pulse is turned off	[26,28,30]
	**ChRWT**_**2**_	9.35×10^-5^			
	**ChETA**	1×10^-3^			
	**ChRET/TC**	3.85×10^-4^			
**τ**_**ChR2**_	**ChRWT**_**1**_	6.32	ms^-1^	Activation time of the ChR2 ion channel	[26,28,30]
	**ChRWT**_**2**_	0.50			
	**ChETA**	1.59			
	**ChRET/TC**	0.36			
**g**_**1**_	**ChRWT**_**1**_	0.11	mS/cm^2^	Maximum conductance of the ChR2 ion channel in the o_1_ state	[26,28,30]
	**ChRWT**_**2**_	0.10			
	**ChETA**	0.88			
	**ChRET/TC**	0.56			
**γ**	**ChRWT**_**1**_	0.03	~	Ratio of maximum conductance of the ChR2 ion channel in the o_2_ and o_1_ state $\left(\frac{g_{2}}{g_{1}}\right)$	[26,28,30]
	**ChRWT**_**2**_	0.02			
	**ChETA**	0.01			
	**ChRET/TC**	0.02			
**E**_**ChR2**_	**All variants**	0	mV	Reversal potential of ChR2	[38]

The components of the **G** matrix are generally determined by the rate parameters of the deterministic part of the SDE, which lead to a state-dependent multiplicative noise in the system [32,44]. In calcium dynamics, the source of stochasticity is mainly attributed to the aggregation of a small number of IP_3_R channels in clusters, which leads to the formation of intra-cluster Ca^2+^ blips (resulting from random opening of a single IP_3_R) or Ca^2+^ puffs and sparks (from the concurrent opening of a large number of IP_3_Rs within a cluster). When coupled with other clusters, synchronized Ca^2+^ puffs can originate global Ca^2+^ events in the cell [32]. Several schemes have been developed to model the stochastic dynamics of IP_3_Rs in different cell types, ranging from models incorporating detailed Markov Chain processes, to those adapting Fokker-Planck approximations (or their equivalent Langevin equations) of the Markov approaches [refer to ref. [45] for a review of the deterministic and stochastic models of IP_3_Rs].

In this study, we adopted the Langevin approach outlined in ref. [43] for the dynamics of open IP_3_R inactivation gates in Eq 1, where the associated noise is a Gaussian white noise with zero mean and the variance ($\sigma_{h}^{2}$) depending on the levels of [Ca^2+^]_c_, [IP_3_], h, and the number of IP_3_Rs in a cluster. We have previously estimated the noise level in the equations for [IP_3_], [Ca^2+^]_c_, and [C_o_] in astrocytes as additive, uncorrelated Wiener processes with zero mean and a constant variance ($\sigma_{i}^{2};i=[{\mathrm{Ca}}_{c}^{2+},{\mathrm{IP}}_{3},C_{o}]$) [35] using the local linearization (LL) filter [46] (Table 1). These processes account for potential sources of stochasticity other than the dynamics of the IP_3_R channels. We further assume that the dynamics of ChR2 gating is only deterministic, i.e., $\sigma_{o_{1}}=\sigma_{o_{2}}=\sigma_{c_{2}}=\sigma_{s}=0$.

The dynamics of the free cytosolic calcium concentration is given by:

$$d{\left[{\mathrm{Ca}}^{2+}\right]}_{c}=(\left[\lambda (J_{{\mathrm{IP}}_{3}R}+J_{\mathrm{Leak}}‐J_{\mathrm{SERCA}})+\varepsilon (J_{\mathrm{in}}+J_{\mathrm{CCE}}‐J_{\mathrm{PMCA}}+J_{\mathrm{ChR}2})\right]/\left(1+\theta \right))\mathrm{dt}+\sigma_{{\mathrm{Ca}}_{c}^{2+}}dw_{{\mathrm{Ca}}_{c}^{2+}},$$ (2)

where θ = b_t_K/([Ca^2+^]_c_+K)^2^ is the buffering factor; b_t_ is the total buffer protein concentration; and K is the buffer rate constant ratio [40].

The efflux of Ca^2+^ from the ER into the cytosol via the IP_3_R channel can be described as:

$$J_{{\mathrm{IP}}_{3}R}=\alpha_{1}v_{1}m_{\infty }^{3}n_{\infty }^{3}h^{3}({\left[Ca^{2+}\right]}_{\mathrm{ER}}‐{\left[Ca^{2+}\right]}_{c}),$$ (2.1)

where

$${\left[Ca^{2+}\right]}_{\mathrm{ER}}=\frac{{(C}_{o}‐{\left[Ca^{2+}\right]}_{c})}{\alpha_{1}}$$ (2.2)

$$m_{\infty }=\frac{\left[{\mathrm{IP}}_{3}\right]}{\left([{\mathrm{IP}}_{3}]+d_{1}\right)}$$ (2.3)

$$n_{\infty }=\frac{{\left[Ca^{2+}\right]}_{c}}{\left({\left[Ca^{2+}\right]}_{c}+d_{5}\right)}$$ (2.4)

The leak of Ca^2+^ ions from ER into the cytosol is modeled as:

$$J_{\mathrm{Leak}}=v_{2}({\left[Ca^{2+}\right]}_{\mathrm{ER}}‐{\left[Ca^{2+}\right]}_{c})$$ (2.5)

A hill-type kinetic model describing the activity of the SERCA pump is given by:

$$J_{\mathrm{SERCA}}=V_{\mathrm{SERCA}}\frac{{\left({\left[Ca^{2+}\right]}_{c}\right)}^{2}}{{\left({\left[Ca^{2+}\right]}_{c}\right)}^{2}+{\left(K_{p}\right)}^{2}}$$ (2.6)

The flux through SOC channels is described using the following equation:

$$J_{\mathrm{CCE}}=\frac{x_{\mathrm{CCE}}{\left(h_{\mathrm{CCE}}\right)}^{2}}{{\left({\left[Ca^{2+}\right]}_{\mathrm{ER}}\right)}^{2}+{\left(h_{\mathrm{CCE}}\right)}^{2}}$$ (2.7)

Ca^2+^ extrusion across the PM via PMCA is given by:

$$J_{\mathrm{PMCA}}=k_{\mathrm{out}}{\left[Ca^{2+}\right]}_{c}$$ (2.8)

PLC_δ1_-mediated IP_3_ changes in the cell is described as:

$$d[{\mathrm{IP}}_{3}]=(X_{IP_{3}}+J_{\mathrm{PL}C_{\delta 1}}‐K_{IP_{3}}[{\mathrm{IP}}_{3}])\mathrm{dt}+\sigma_{{\mathrm{IP}}_{3}}dw_{{\mathrm{IP}}_{3}},$$ (3)

where $X_{IP_{3}}$ denotes the basal rate of IP_3_ production in the cell. PLC_δ1_ activity is described with a Hill’s kinetic model as:

$$J_{\mathrm{PL}C_{\delta 1}}=v_{\delta }\frac{{{\left[{\mathrm{Ca}}^{2+}\right]}_{c}}^{2}}{{{\left[{\mathrm{Ca}}^{2+}\right]}_{c}}^{2}+{K_{\mathrm{\delta Ca}}}^{2}}$$ (3.1)

The dynamics of the fraction of open inactivation IP_3_R gates is given by:

$$\mathrm{dh}=[\alpha_{h}(1‐h)‐\beta_{h}h]\mathrm{dt}+\sigma_{h}(t,x,p)dw_{h},$$ (4)

where the opening (α_h_) and closing (β_h_) rates are defined as:

$$\alpha_{h}=\frac{ad_{2}([IP_{3}]+d_{1})}{[IP_{3}]+d_{3}}$$ (4.1)

$$\beta_{h}=a{\left[Ca^{2+}\right]}_{c}$$ (4.2)

and

$$\sigma_{h}^{2}(t,x,p)=\left[\alpha_{h}(1‐h)+\beta_{h}h\right]/N,$$ (4.3)

where N is the number of IP_3_Rs within a cluster.

The total free Ca^2+^ in the cell is modeled as:

$$dC_{o}=(\varepsilon (J_{\mathrm{in}}+J_{\mathrm{CCE}}‐J_{\mathrm{PMCA}}+J_{\mathrm{ChR}2})/\left(1+\theta \right))\mathrm{dt}{+\sigma }_{C_{o}}dw_{C_{o}},$$ (5)

with a zero-mean Gaussian noise component with a constant variance $\sigma_{C_{o}}^{2}$.

The open and closed gating dynamics of ChR2 are given by:

$$do_{1}={(p}_{1}sc_{1}‐(G_{d_{1}}+e_{12})o_{1}+e_{21}o_{2})\mathrm{dt}{+\sigma }_{o_{1}}dw_{o_{1}}$$ (6)

$$do_{2}=(p_{2}sc_{2}+e_{12}o_{1}‐(G_{d_{2}}+e_{21})o_{2})\mathrm{dt}{+\sigma }_{o_{2}}dw_{o_{2}}$$ (7)

$$dc_{2}=(G_{d_{2}}o_{2}‐(P_{2}s+G_{r})c_{2})\mathrm{dt}{+\sigma }_{c_{2}}dw_{c_{2}}$$ (8)

$$\mathrm{ds}=\left(\frac{\left(S_{0}(t)‐s\right)}{\tau_{\mathrm{ChR}2}}\right)\mathrm{dt}{+\sigma }_{S}dw_{S}$$ (9)

The existence of ChR2 in open and closed states satisfies the following algebraic condition:

$$c_{1}+c_{2}+o_{1}+o_{2}=1$$ (10)

The current generated through ChR2 is given by:

$$I_{\mathrm{ChR}2}=A_{m}g_{1}(o_{1}+\gamma o_{2})G(V_{m})(V_{m}‐E_{\mathrm{ChR}2}),$$ (11)

where G(V_m_) is the voltage dependent rectification function determining the shape of ChR2 photocurrent with changing V_m_. The resultant flux through ChR2 is then derived as

$$J_{\mathrm{ChR}2}=\frac{I_{\mathrm{ChR}2}}{F{\mathrm{vol}}_{\mathrm{cyt}}z_{{\mathrm{Ca}}^{2+}}}$$ (11.1)

Although ChR2 is a non-selective cation channel, in this study, we assume that J_ChR2_ is solely a calcium flux, as calcium dynamics is one of the most prominent modes of signaling in astrocytes.

### Quantification of astrocytic activity

**Fig 2:**
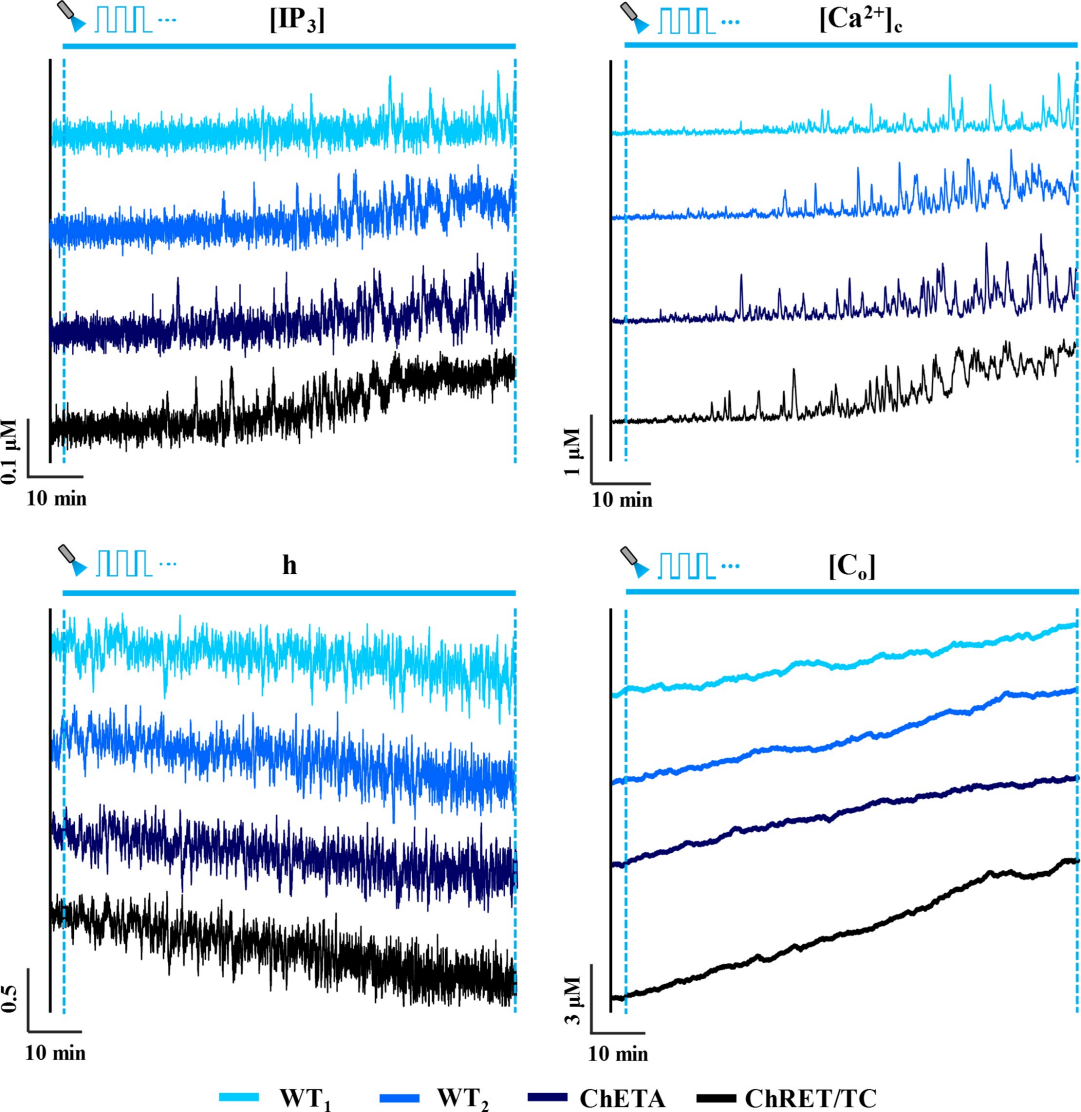
Response of ChR2 variants to light stimulation. Representative traces (90 minutes in duration) of IP_3_ concentration ([IP_3_]), cytosolic calcium concentration ([Ca^2+^]_c_), fraction of open inactivation IP_3_R gates (h), and total calcium concentration ([C_o_]) for an astrocyte expressing various ChR2 variants (wild type 1 (WT_1_), wild type 2 (WT_2_), ChETA and ChRET/TC) in response to light stimulation (T = 2 s, δ = 20% (0.4 s), unit amplitude). The blue horizontal solid line indicates the stimulation window.

**Fig 3:**
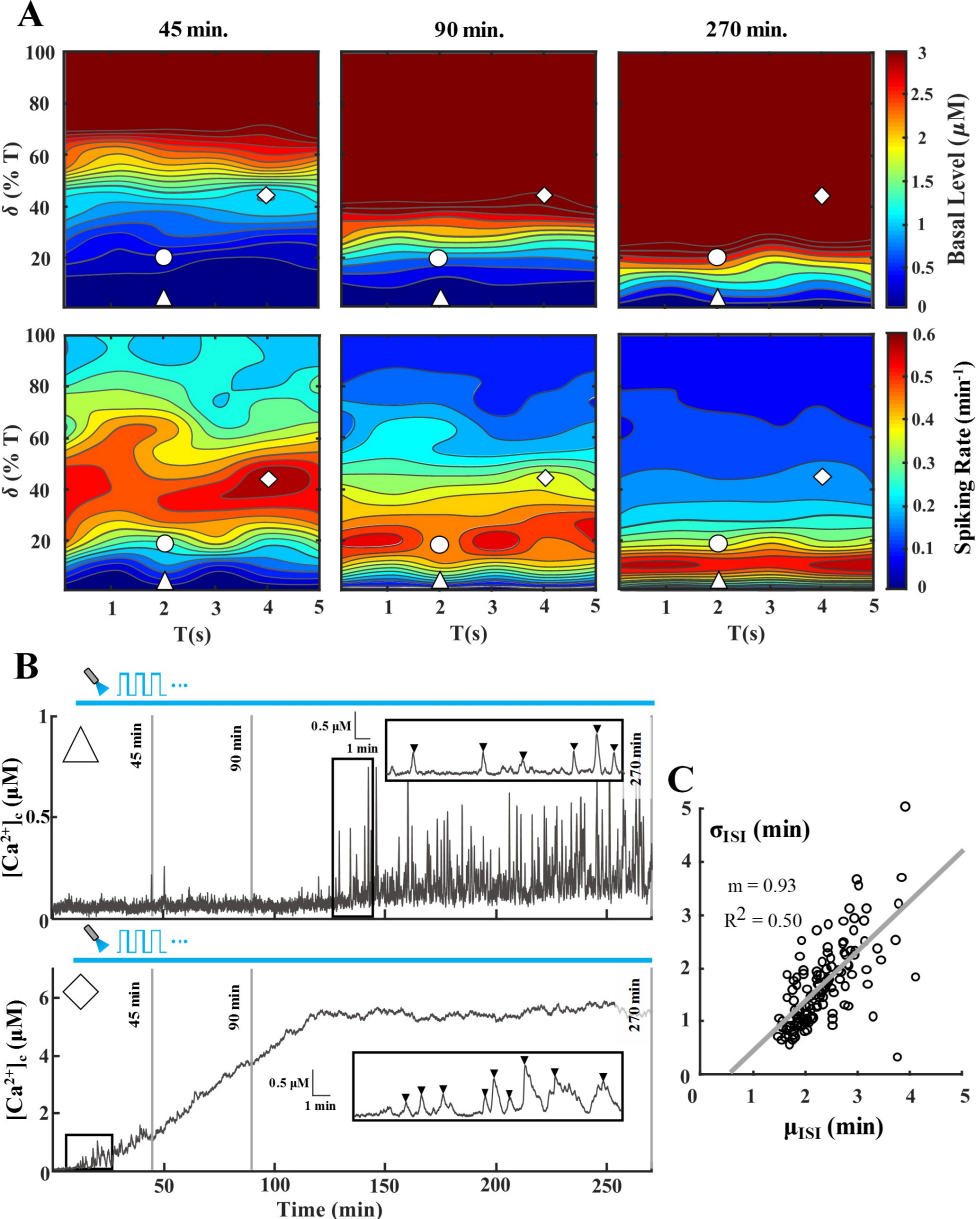
Response of a ChETA-expressing astrocyte to various light stimulation paradigms. Simulations were conducted to evaluate astrocytic Ca^2+^ response to different paradigms ranging from T = 1–5 s and δ = 0–100% of T (trials = 5). **A)** Heat maps of Ca^2+^ basal level (top panel) and spiking rate (bottom panel) for the T-δ combinations assessed. Each column depicts basal level and spiking rate heat maps up to a certain point, i.e. 45, 90, and 270 minutes, to determine the cell response as light stimulation progresses from short-term to long-term. Scale bars for heat maps of basal level and spiking rate were capped to 3 μM and 0.6 spikes/min, respectively. The △ and ◊ symbols correspond to T-δ combinations that result in the traces of panel B. **B)** Representative [Ca^2+^]_c_ traces corresponding to T-δ combinations highlighted in A [Top trace (△): T = 2 s, δ = 1% (0.02 s); Bottom trace (◊): T = 4 s, δ = 45% (1.8 s)]. The inset of each trace highlights a 15-minute section to show the detected Ca^2+^ spikes. For spike detection, a threshold of 0.2 μM above the basal level was utilized. Each vertical solid grey line denotes the time points corresponding to heatmaps of panel A. **C)** Average (μ_ISI_) vs. standard deviation of ISI (σ_ISI_) where each point corresponds to a single simulated 270-min [Ca^2+^]_c_ trace. Solid line shows the linear fit between μ_ISI_ and σ_ISI_ values. Throughout the manuscript, △, ○, and ◊ represent paradigms resulting in low, medium, and high Ca^2+^ spiking during short-term (45 min) stimulation of astrocytes, respectively.

Throughout this manuscript, we calculated the light-evoked mean spiking rate and basal level of [Ca^2+^]_c_ as a measure of astrocytic activity. For spiking rate calculations, we first removed the trend of simulated calcium traces, i.e., ChR2-induced steady rise in the baseline levels as observed in the traces of Figs 2 and 3. Using a threshold of 0.2 μM, calcium spikes were detected from the baseline-corrected traces and used to compute the mean spiking rate over the entire duration of stimulation. This threshold was chosen to exclude small-amplitude Ca^2+^ fluctuations from the calculation of the mean spiking rate. Light-induced elevations in the Ca^2+^ basal levels were calculated at the end of the stimulation window. These two measures were compared to study the effect of different light stimulation paradigms, i.e., varying combinations of T and δ values, as well as model parameters, on the response of optogenetically-stimulated astrocytes.

### Sensitivity analysis

A global sensitivity analysis was performed to assess the sensitivity of the single cell model output, i.e., Ca^2+^ spiking rate and basal level, to variation in parameters determining the magnitude of ChR2 photocurrent and those describing the Ca^2+^ buffering kinetics. Each parameter was allowed to vary around its control value within a lower and upper bound (Table 2), and the Latin hypercube sampling (LHS) method with uniform distribution was used to select 1000 random parameter sets to perform the sensitivity analysis [47,48]. The upper and lower bounds were selected such that the range encompasses reported values of parameters for different variants of ChR2. The single cell model was solved for each parameter set, and the partial rank correlation coefficient (PRCC) for each parameter was then computed to determine the magnitude of the parameter influence (positive or negative) on the desired model output. A 95% confidence interval was chosen to determine if the exerted influence is statistically significant.

**Table 2:** Range of parameters for global sensitivity analysis.

Parameter	Range	Unit
**ChR2 parameters**		
**p**_**1**_	51.28−1.50×10^2^	ms^-1^
$G_{d_{1}}$	8.16−5.47×10^2^	ms^-1^
**e**_**12**_	163.52−1.93×10^4^	ms^-1^
**e**_**21**_	4.00−1.92×10^3^	ms^-1^
**p**_**2**_	10.00−7.69×10^1^	ms^-1^
$G_{d_{2}}$	56.32−1.81×10^2^	ms^-1^
**G**_**r**_	7.48×10^-2^−1.20	ms^-1^
**τ**_**ChR2**_	2.89×10^-4^−7.6×10^-3^	ms^-1^
**γ**	1.13×10^-2^−3.66×10^-2^	~
**g**_**1**_	7.84×10^-2^−1.05	mS/cm^2^
**Buffering Parameters**		
**b**_**t**_	160−240	μM
**K**	16−24	μM

### Astrocytic network model

To study the network-wide response of astrocytes to light stimulation, we incorporated Ca^2+^ and IP_3_ permeable gap junctions between cells in a network of astrocytes. Fluxes:

$$J_{{\mathrm{gj}}_{{\mathrm{Ca}}_{i}^{2+}}}=\sum_{k}D_{{\mathrm{Ca}}^{2+}}([{{\mathrm{Ca}}^{2+}]}_{c_{i}}‐[{{\mathrm{Ca}}^{2+}]}_{c_{k}})$$ (12)

$$J_{{\mathrm{gj}}_{{{\mathrm{IP}}_{3}}_{i}}}=\sum_{k}D_{{\mathrm{IP}}_{3}}([{{\mathrm{IP}}_{3}]}_{i}‐[{{\mathrm{IP}}_{3}]}_{k})$$ (13)

represent the flow of Ca^2+^ and IP_3_, respectively, from astrocyte ‘i’ in the network to its neighboring cells (indicated by index k). Local and global calcium events are simulated upon focal light stimulation of astrocytes, i.e., a region in the center of the network. Simulations were performed with varying levels of Ca^2+^ coupling coefficient, $D_{{\mathrm{Ca}}^{2+}}$, in the presence and absence of Ca^2+^ buffering. Calcium responses in the network, i.e., the basal levels and spiking rates, were fitted with a symmetrical 2D Gaussian profile (H(ζ)) to quantify the peak Ca^2+^-basal/spiking (h_max,ζ_), as well as the magnitude of the spread of Ca^2+^-basal/spiking activity (σ_ζ_) within the network:

$$H(\zeta )=h_{\min,\zeta }+\frac{\left(h_{\max,\zeta }‐h_{\min,\zeta }\right)}{2\pi \sigma_{\zeta }^{2}\;\exp(‐\frac{{\left(x‐x_{0}\right)}^{2}+{\left(y‐y_{0}\right)}^{2}}{2\sigma_{\zeta }^{2}})},$$ (14)

where ζ represents calcium basal level or mean spiking rate, h_min,ζ_ is the minimum value of the ζ in the network, x_0_ and y_0_ are the indices of the astrocyte at the center, and x and y are the location of cells away from the origin in both directions. The effect of calcium buffering, varying levels of $D_{{\mathrm{Ca}}^{2+}}$, and the number of cells expressing ChR2 on both the peak and spread of basal/spiking activity was investigated.

All simulations were performed in MATLAB using the Local Linearization method described in refs. [46,49] with an integration step size of 0.1 ms. A listing of all parameters and their descriptions is provided in Tables 1 and 2. The codes associated with this study can be downloaded from http://web.eng.fiu.edu/jrieradi/CaAnalysisCode/.

## Results

### Effect of ChR2 variants on the light-evoked response of astrocytes

Representative traces for the time evolution of state variables of Eq 1 for astrocytes expressing different variants of ChR2 are illustrated for 90 minutes of light stimulation (Figs 2 and S1). Simulations reveal a progressive increase in the spiking activity of cytosolic calcium in all variants with the duration of stimulus. Apparent in the traces is a lag between the onset of light stimulation and regions with more regular spiking activity, likely reflecting the time required for [Ca^2+^]_c_ to reach sufficient levels to activate IP_3_R channels. Traces show a steady increase in the total calcium content of the cell, and conversely, a decreasing trend in the fraction of open IP_3_R inactivation gates (h). The [IP_3_] levels, however, show a rather steady profile for WT and ChETA variants, and predict only a slight increase towards the end of the stimulation window for ChRET/TC. This increase can be attributed to the elevated basal [Ca^2+^]_c_ in ChRET/TC expressing astrocytes, activating the production of IP_3_ via the PLC_δ1_ pathway ([Ca^2+^] at half- maximal activation of PLC_δ1_ is ~0.5 μM, Table 1). Comparison of results across variants suggests that light-induced changes are steepest in astrocytes expressing ChRET/TC. These model predictions are in line with the higher magnitude of the ChR2 flux in ChRET/TC compared to other variants in both transient and plateau regions of the photocurrent during the light stimulus (S2 Fig). Furthermore, results suggest that for the simulation time in this figure, the mechanism of light-evoked increased spiking in cytosolic calcium, predominantly in WT or ChETA enabled astrocytes, is mainly through the activation of IP_3_Rs by increases in [Ca^2+^]_c_ rather than elevations in [IP_3_].

### Effect of light stimulation paradigm on single cell model response

**Fig 4:**
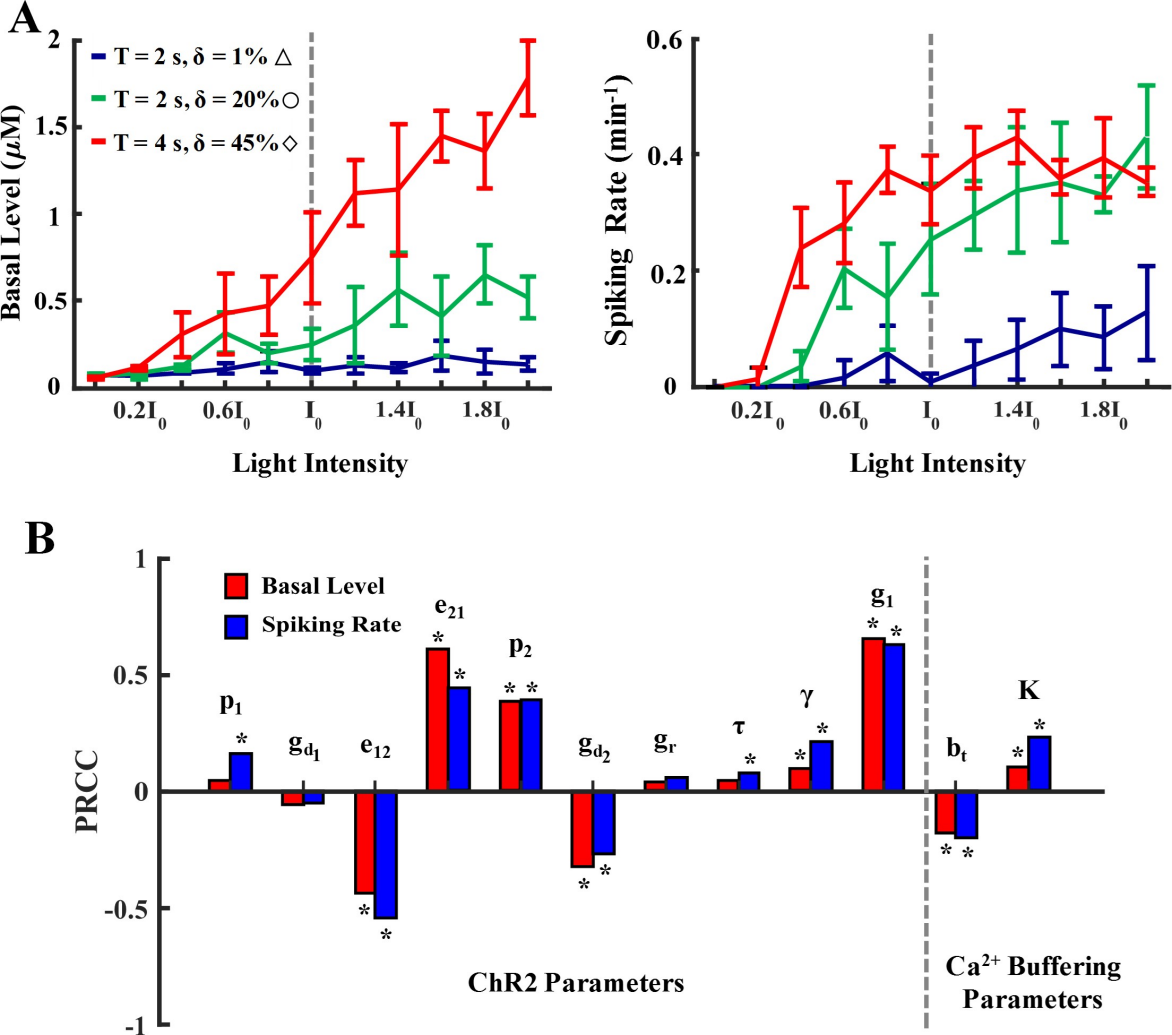
Model sensitivity to light intensity, Ca^2+^ buffering, and ChR2 parameters. **A)** Effect of different light intensities and stimulation paradigms on the basal level and mean spiking rate of a ChETA-expressing astrocyte. Light intensity was varied as a fraction of the control value (I_0_). Stimulation paradigms were selected based on the 45-min spiking rate heat map in Fig 3A for low (△), medium (○), and high (◊) activity regions. Data is shown as mean ± std (trials = 5). **B)** Global sensitivity analysis of the astrocytic Ca^2+^ response to variations in ChR2 and Ca^2+^ buffering parameters during light stimulation (Δ: T = 2 s, δ = 1% (0.02 s)). Parameters were allowed to vary within a range (Table 2) and 1000 parameter sets were selected using Latin Hypercube Sampling (LHS) with uniform distribution. The partial rank correlation coefficient (PRCC) of each parameter was calculated as a measure of their effect on the basal level (red) and spiking rate (blue) of a ChR2-expressing astrocyte. * denotes statistically significant (p < 0.05) positive or negative influence.

Heat maps (Fig 3A) show the effect of various light stimulation paradigms on the spiking rate and Ca^2+^ basal level in ChETA-expressing astrocytes for different stimulation times, i.e., 45, 90, and 270 minutes (for other ChR2 variants refer to S3 Fig). Simulations show that as the stimulus duration increases, the Ca^2+^ basal level rises steadily due to a net Ca^2+^ entry into the cytosol (note the upward trend of [C_o_] in the traces of Fig 2). This effect is demonstrated in representative traces of Fig 3B, where depending on the T-δ combination, [Ca^2+^]_c_ may reach supraphysiological, toxic levels which bring the cell outside of the window for regular spiking (i.e., [Ca^2+^]_c_ window for IP_3_R-mediated Hopf bifurcation; notice the bottom trace of Fig 3B). Consequently, T-δ combinations that elicit high spiking activity in astrocytes during short-term stimulation (45 min) will transition into regions with medium and low activity as the stimulus progresses (notice the ◊ symbol in spiking rate heat maps, bottom panel of Fig 3A). Conversely, as seen in the top trace of Fig 3B, combinations with low levels of spiking in short-term stimulations might transition into regions eliciting high calcium activation, with basal levels within the physiological range, as the stimulation progresses. Thus, to remain within optimal spiking and basal [Ca^2+^]_c_ levels, one might choose stimulus waveforms based on the desired stimulation duration. It should be noted, however, that the predicted behavior is also a consequence of Ca^2+^ buffering capacity of the cell, light intensity, and parameters determining the shape and magnitude of the ChR2 photocurrent (Fig 4).

To quantify the regularity of light-evoked calcium spiking in the astrocyte model, we demonstrate the σ_ISI_−μ_ISI_ relationship for the inter-spike interval (ISI) values, i.e., the time between subsequent calcium spikes, of simulations in Fig 3A. The average and standard deviation of ISI of the [Ca^2+^]_c_ traces are represented by μ_ISI_ and σ_ISI_, respectively. This analysis has been performed for different cell types, including astrocytes, during spontaneous as well as IP_3_-induced calcium oscillations [31,32]. In agreement with these experimental observations, results from our simulations also show a positive and linear correlation between μ_ISI_ and σ_ISI_ values in the astrocytic calcium spiking during light stimulation (Fig 3C). Regions with Ca^2+^ levels outside of the predicted range for sustained oscillations, i.e., regions with either low or supraphysiological Ca^2+^ basal levels, result in infrequent and irregular spiking with higher μ_ISI_ and σ_ISI_ values.

### Sensitivity to model parameters

Simulations predict salient features of light-induced responses in our model astrocyte, i.e., regions with elevated calcium spiking and a steady rise in the Ca^2+^ basal level. Results in Fig 4A show the effect of light intensity, or equivalently the amplitude of the waveform, on Ca^2+^ response of ChETA-expressing astrocytes for three distinct T-δ combinations. These paradigms correspond to regions with low (△), medium (○) and high (◊) spiking activity in the 45-minute stimulation heatmap of Fig 3A, respectively. For all combinations, the Ca^2+^ basal level shows an upward trend with increasing light intensity (with varying slopes), indicating a larger Ca^2+^ influx through ChR2. Similar trends are observed for spiking rates in the case of low and medium T-δ combinations. For the stimulus paradigm with highest spiking under control conditions (I_0_), the spiking rate plateaus over a range of intensity values and follows a declining trend upon further increase in the light intensity. This indicates that the associated increase in the basal level leads to a decrease in the firing rate of astrocytes as cytosolic calcium levels exit the region where regular oscillations can occur. These model predictions are similar to experimental observations of monotonic increase in the firing of ChR2-positive neurons stimulated with increasing light intensities [50].

We further performed a global sensitivity analysis to evaluate the effect of parameters determining the dynamics of ChR2 photocycle and buffering capacity on the astrocytic Ca^2+^ responses for a low light stimulation paradigm (Fig 4B). Parameters that exerted statistically significant influence on the desired model output are marked with an asterisk. Results indicate differential effects of model parameters on Ca^2+^ response of the astrocyte to light stimulation. For instance, increasing the maximum conductance of ChR2 in o_1_ state (g_1_) is positively correlated with both spiking rate and basal levels, as the elevated conductance results in higher magnitudes of Ca^2+^ influx through the channel resulting in the elevation of basal levels and further activation of IP_3_Rs. Similarly, e_21_ (the rate of transition from o_2_ to o_1_) has a positive correlation with both measures, while the rate of the reverse reaction (e_12_) is shown to be negatively correlated to the model output. These results suggest that by transitioning the state of ChR2 from o_2_ to o_1_, both spiking rate and basal level of Ca^2+^ will likely increase. This model prediction can be attributed to the higher conductance of the channel in the o_1_ state, the transient region of the ChR2 photocurrent in S2 Fig, compared to o_2_ state, the plateau region. Sensitivity analysis of the Ca^2+^ buffering parameters demonstrates that with increasing buffering capacity, i.e., increasing total buffer concentration (b_t_) or reducing the affinity of buffer proteins to Ca^2+^ (K), both basal Ca^2+^ level and mean spiking rate of astrocytes will expectedly decrease.

### Network-wide astrocytic response to light stimulation

**Fig 5:**
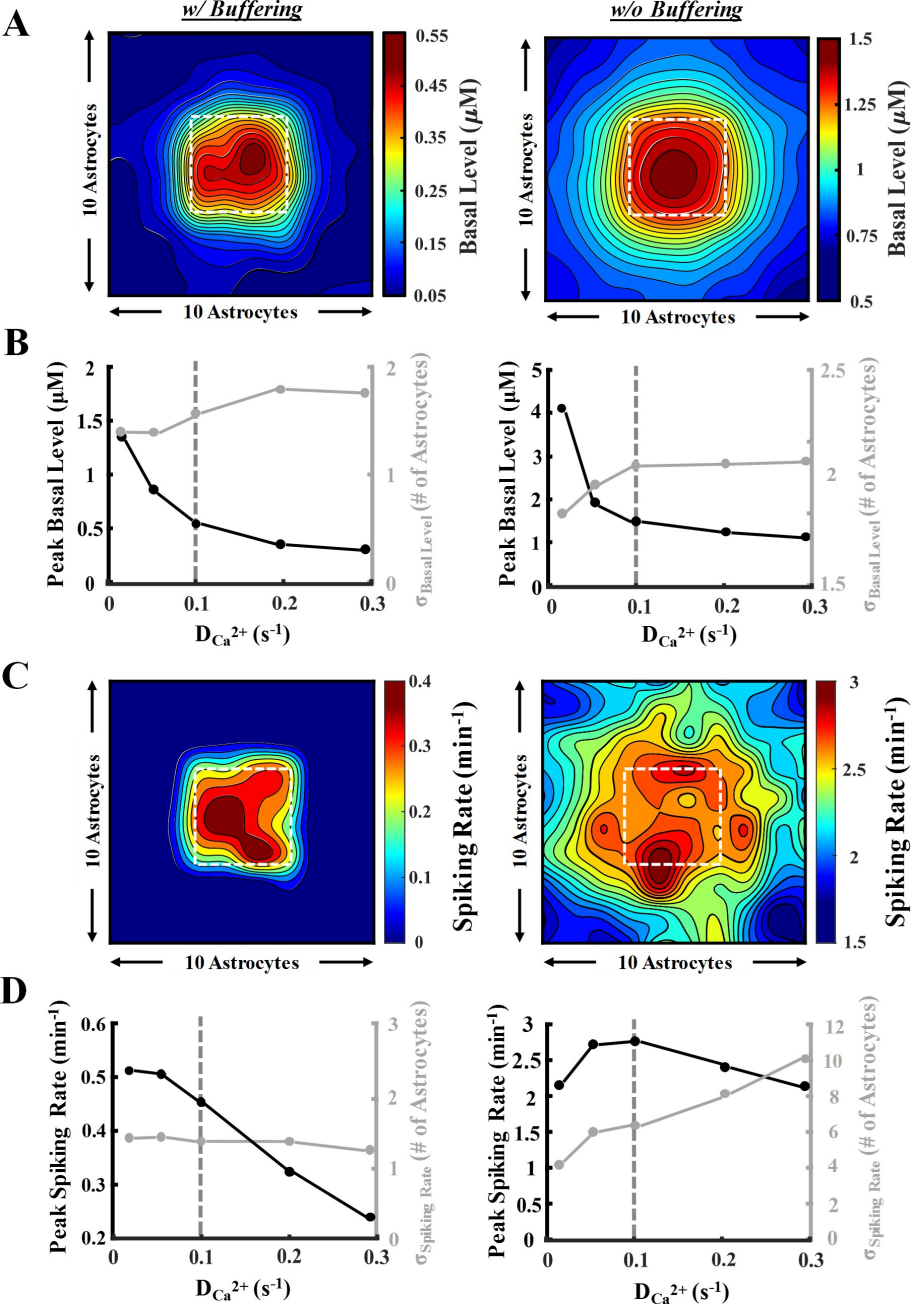
Network-wide astrocytic Ca^2+^ response to light stimulation. A 10-by-10 network of astrocytes was employed to analyze the response of cells when the central 4-by-4 astrocytes (white square in heatmaps of panels A and C) were stimulated (◊: T = 4 s, δ = 45% (1.8 s), 45 min). See the network organization schematic in Fig 1. Simulations were conducted in the presence (left panel) and absence (right panel) of Ca^2+^ buffering with varying gap junctional Ca^2+^ coupling coefficient ($D_{{\mathrm{Ca}}^{2+}}$). A symmetric 2D Gaussian fit was utilized to quantify the response, i.e., peak and magnitude of the spread from the stimulated region. **A)** Heat maps of network-wide light stimulation-induced Ca^2+^ basal levels. **B)** Plots of the peak basal level and σ_basal-level_ obtained from the Gaussian fit with varying $D_{{\mathrm{Ca}}^{2+}}$ values. Vertical dashed grey line denotes the $D_{{\mathrm{Ca}}^{2+}}$ value used to generate the heat maps in panels A and C. **C)** Heat maps of network-wide spiking rate response corresponding to basal levels in panel A. For spike classification, a threshold of 0.2 μM above the basal level was selected. **D)** Plots for the peak spiking rate and σ_spiking rate_ with varying $D_{{\mathrm{Ca}}^{2+}}$ values.

A 10-by-10 network of gap-connected astrocytes was modeled in Fig 5 and S1 Movie. Light stimulation (T = 4s, δ = 45%; $D_{{\mathrm{Ca}}^{2+}}$ = 0.1 s^-1^) was applied to a central region (the highlighted box in Fig 5A), and simulations were performed for 45 minutes. Fig 5A heatmaps demonstrate the resulting basal Ca^2+^ level in the network after light stimulation, with and without the inclusion of Ca^2+^ buffering. Under both conditions, results show increased basal levels in the stimulated region and the propagation of calcium to unstimulated cells. Basal levels reached in focal and distal astrocytes without buffering were drastically higher compared to those with buffering. In the presence of Ca^2+^ buffering, only the area in close vicinity of the stimulated region exhibits an increase in the basal level; whereas, in the absence of buffering, even the cells farthest from the stimulation region undergo an increase in calcium. This suggests that buffering reduces the diffusion range of Ca^2+^ within the network, thereby limiting propagation. Given the focal and centered stimulation of the network, the distribution of calcium can be suitably quantified using a 2D Gaussian fit (Eq 14). In Fig 5B, the peak basal level, along with the spread from the center (in terms of number of astrocytes), are quantified with varying $D_{{\mathrm{Ca}}^{2+}}$ values. In both cases, an intuitive decrease in peak basal level coupled with an increase in the spread is observed as $D_{{\mathrm{Ca}}^{2+}}$ increases. Both trends plateaued as $D_{{\mathrm{Ca}}^{2+}}$ values reached higher than 0.1 s^-1^.

Similar responses are observed in the mean spiking rate of the astrocytic network upon light stimulation (Fig 5C). Inclusion of calcium buffering limited the spiking activity of astrocytes only in regions immediately surrounding the stimulated region. On the contrary, without calcium buffering, the spiking activity propagated to unstimulated areas and resulted in higher mean spiking rate values. Analysis of network-wide peak spiking rate and spread with buffering in Fig 5D (left panel) indicates a decreasing trend of peak spiking with $D_{{\mathrm{Ca}}^{2+}}$. The propagation of the spiking rate, however, remained almost unchanged over the entire range of $D_{{\mathrm{Ca}}^{2+}}$ values examined. Conversely, when buffering was not included (Fig 5D, right panel), the increase in $D_{{\mathrm{Ca}}^{2+}}$ resulted in an increasing trend in the spread of spiking activity while the peak spiking rate remained at high values throughout. Increasing the number of cells expressing ChR2 in the network also resulted in a steady increase in both spiking rate and calcium basal levels in the network (S4 Fig) when buffering was included. Under no buffer condition, the peak basal level showed a steep incline, while the spread of the basal activity remained constant. Both peak spiking rate and spread showed an upward trend with increased expression level, with peak levels reaching a plateau when higher than 50% of the cells expressed ChR2. Collectively, simulations in Fig 5 highlight the role of calcium buffering in limiting the diffusion of free calcium in the network of astrocytes and thereby reducing both basal and spiking levels of calcium in cells. Additionally, comparison of the network results with those of single cells (Fig 3) reveals that the dispersion of calcium into the neighboring cells through gap junctions drastically reduces the supraphysiological values of calcium concentrations predicted in isolated cells.

## Discussion

The past few decades have witnessed an upsurge in research on understanding the role of astrocytes in brain function. Aside from providing structural support to neurons [51,52] and regulating the ‘excitatory-inhibitory’ neurotransmitter balance [53], the exact contribution of these cells, particularly in processes involving their Ca^2+^ signaling, is unknown and is highly debated [see refs [1–6]]. For instance, astrocytes are thought to be directly involved in neurovascular coupling by sensing neuronal activity and releasing Ca^2+^-mediated vasoactive agents to relax smooth muscle cells of parenchymal arterioles [14,54,55]. However, recent experimental evidence has thrown the validity of this assumption into disarray by questioning astrocytes’ involvement, and the extent of their influence, in the onset of the hyperemic response [16,56,57]. Another controversy involving astrocytes is their potential role in modulating neuronal activity via Ca^2+^-mediated release of gliotransmitters. While several groups present evidence for the active involvement of gliotransmission in neuromodulation, others argue that this process is not likely to occur under physiological conditions [4,5,58]. Ca^2+^ homeostasis is also dramatically altered in astrocytes under several pathological conditions and during reactive gliosis [59]. These include elevated Ca^2+^ levels, as well as intercellular waves in epilepsy [60], Alzheimer’s disease [34,61], and spreading depression [62,63]. Despite the involvement of astrocytes in these multi-mechanistic phenomena, differentiating their specific contributions from concurrent neuronal activity has remained one of the most enduring challenges in the field. Thus, selectively targeting astrocytes, and their Ca^2+^ signaling, using advanced techniques like optogenetics can provide assistance in resolving the above controversies and help find answers to their exact roles in health and disease. The mathematical modeling framework outlined in our study is a step in this direction in providing a tool for experimentalists to precisely achieve desired astrocytic Ca^2+^ levels to examine their role in the abovementioned phenomena.

### Salient features of light-evoked calcium signaling in astrocytes

Simulation results predict that calcium dynamics in astrocytes, as seen in experimental studies [33,64,65], can be heavily regulated by light-induced activation of ChR2. Consistent in all simulations performed in this study, upon light activation, astrocytes underwent increases in their basal calcium level and exhibited changes in the spiking activity (Figs 2 and 3). Our results demonstrate that the extent of the rise in calcium (reflecting the higher magnitude of entry through ChR2 compared to the rate of scavenging by PMCA, SERCA, and buffer proteins) is largely dependent on the specifications of the laser stimulus, i.e., T-δ combination and light intensity, as well as parameters determining the shape of the ChR2 photocurrent (Figs 3 and 4). Additionally, whether astrocytes show high or low spiking activity is also contingent upon the duration of light stimulation. As such, T-δ combinations with high spiking activity of single cells in short-term stimulations may transition to regions with medium or low spiking in longer durations, or vice versa (Fig 3B). These results emphasize the importance of choosing an ‘ideal’ T and δ combination for the desired short-term or long-term astrocytic activity. An inaccurate selection of these combinations could prompt astrocytes to an unphysiological Ca^2+^ signaling regime, which might be detrimental for the health of the cells. Whether these model predictions are physiologically accurate needs to be validated against experimental observations for both short and long-term activation of ChR2. When coupled with other astrocytes in a network, however, the dispersion of calcium to neighboring cells dramatically reduced the basal levels reached in the stimulated region (Fig 5A), with values depending on the magnitude of the calcium and IP_3_ coupling coefficients in the network. This indicates that experimental design for optogenetic stimulation of a network of astrocytes, e.g., *in vivo* recordings, cannot be solely based on predictions drawn from single cells, and that network activity depends on ChR2 expression level (S4 Fig).

### Engineering of application-based ChR2 variants

Several research groups have engineered ChR2 variants with distinct characteristics, e.g. enhanced conductance, sensitivity, and faster recovery kinetics [26,28] for specific applications in excitable cells. Results of our study can be useful in the development of future ChR2 constructs for eliciting desired activity targeting astrocytes. More specifically, results of our sensitivity analysis (Fig 4B) indicated that the kinetics of ChR2 photocycle significantly affect the Ca^2+^ spiking rate and basal levels. Intuitively, directing ChR2 to the open states (o_1_ and o_2_) from the closed states (c_1_ and c_2_) leads to an increase in astrocytic activity in response to light stimulation. For instance, decreasing $G_{d_{1}}$ and $G_{d_{2}}$ facilitates the existence of ChR2 in the open states as they are negatively correlated to both basal level and spiking rate. Also, an increase in p_2_ drives the system to the open state and is positively correlated to both measures. However, less intuitively, for the simulations performed in our study, increased astrocytic activity was achieved when ChR2 resided more in the o_1_ state as compared to the o_2_ state. This can be observed as an increase in e_21_ and a decrease in e_12_ leading to the existence of ChR2 in o_1_ (see Fig 1 ChR2 photocycle). The shape of the photocurrents in S2 Fig also confirms that the channel has the highest flux in the o_1_ state (the transient phase). Collectively, these results suggest that tailoring new ChR2 constructs such that the photocurrent is directed mainly towards the o_1_ state can enhance the astrocytic activity. The same analysis can be performed under different stimulation paradigms and for varying durations of stimulus.

### Model limitations and future directions

ChR2 is a non-selective cation channel, with varying permeability for Na^+^, K^+^, Ca^2+^, and H^+^ across different variants [24,25]. Cationic entry has shown to induce membrane depolarization which can activate voltage-gated calcium channels, although their functional role in astrocytes is a subject of ongoing debate [58], and result in further influx of calcium, potentially activating large conductance calcium activated potassium currents [66]. Activation of these channels change the membrane potential of the cell and can thus affect the magnitude of the ChR2 photocurrent, since ChR2 is V_m_-sensitive. Our model does not include the dynamics of other major ionic species and does not account for the abovementioned dependencies on the membrane potential. We have also not explicitly accounted for other intracellular compartments, e.g. microdomains and mitochondria, involved in the calcium dynamics of astrocytes. In a recent study [67], it was demonstrated that inclusion of microdomains and mitochondria compartments reduced the calcium and IP_3_ levels required for the activation of IP_3_Rs in non-excitable cells and can potentially affect the ChR2-induced calcium signaling in our model. Another limitation of the model is that, in this study, we adopted the Langevin approach outlined in ref. [43] for the implementation of stochasticity in the dynamics of IP_3_R channels and have not accounted for the detailed diffusion of calcium ions within and between IP_3_R clusters. This would require a system of partial differential equations as demonstrated in ref. [32]. In this study, we sought to provide a minimalistic theoretical framework which can readily be employed by researchers for the investigation of light induced Ca^2+^ responses in astrocytes. Combination of the presented model with more detailed models as in [42] and [68] where exhaustive geometry and dynamics of various ionic species are accounted for, can enhance our understanding of the intricacies of the behavior of astrocytes and their response to light.

## Supporting information

S1 Fig ChR2 gating dynamics during light stimulation. Representative traces of open state (o_1_ and o_2_) and closed state (c_1_ and c_2_) gating dynamics during light stimulation, corresponding to the 90-min simulation depicted in Fig 2 (wild type 1 (WT_1_), wild type 2 (WT_2_), ChETA. and ChRET/TC). A 30-second segment of each trace is shown to highlight details. Prior to light stimulation, all variants reside in the c_1_ state. Once stimulation is initiated, the variants reside in different states at varying levels. Within the open states, they mainly reside in o_2_. Solid horizontal blue line corresponds to the period during which the light stimulation was on.
(TIF)

S2 Fig Comparison of the ChR2 flux across variants. Simulated ChR2 channel flux (J_ChR2_) in response to a 1-sec pulse stimulation. Solid horizontal blue line corresponds to the period during which the light pulse was on. The solid grey box highlights a transient phase, during which all variants exhibit a brief large-magnitude flux (ChRET/TC > WT_2_ > WT_1_ > ChETA), corresponding to the light-induced transition to the o_1_ state. The dashed grey box shows the plateau phase of the flux, corresponding to the transition and stabilization in the low-magnitude o_2_ state. The plateau phase flux magnitudes are in the order ChRET/TC > ChETA > WT_2_ > WT_1_.
(TIF)

S3 Fig Response of astrocytes expressing different ChR2 constructs to various light stimulation paradigms. Simulations (45 min) were conducted to evaluate astrocytic Ca^2+^ response while expressing various ChR2 constructs. Different stimulation paradigms ranging from T = 1–5 s and δ = 0–100% of T (trials = 5) were applied from 100 seconds, until the end of the simulation. Each column corresponds to an evaluated ChR2 variant, i.e. WT_1_ (left), WT_2_ (center) and ChRET/TC (right). Heat maps of Ca^2+^ basal level (top panels) and spiking rate (bottom panels) for T-δ combinations are depicted. Scale bar for each heat map was capped to 3 μM and 0.6 spikes/min, respectively. For spike detection, a threshold of 0.2 μM above the basal level was utilized. Results show a similar basal level and spiking rate distribution for WT_1_ and WT_2_. However, ChRET/TC shows a smaller region of increased spiking activity coupled with a larger region of T-δ combinations eliciting supraphysiological basal level changes.
(TIF)

S4 Fig Effect of expression heterogeneity on the network-wide response to stimulation. A 10-by-10 network of astrocytes was used to demonstrate the resulting response when the expression of the central 4-by-4 astrocytes (white square in Fig 5A and 5C) is varied [Light stimulation: (◊) T = 4 s, δ = 45% (1.8 s)]. Simulations were conducted for 45 minutes while the percentage of the central astrocytes randomly selected to express ChETA was varied (25, 50, 75, 100%) in the presence (left panel) and absence (right panel) of Ca^2+^ buffering. A total of 5 trials were conducted for each expression level. A symmetric 2D Gaussian fit was used to quantify the response, i.e. peak and magnitude of the spread from the stimulated region. Top row of plots shows the average and standard deviation of peak basal level and σ_basal level_ as a function of expression. Bottom row of plots shows the average and standard deviation of peak spiking rate and σ_spiking rate_ as a function of expression. For spike classification, a threshold of 0.2 μM above basal level was selected. Increasing the number of cells expressing ChR2 in the stimulation region resulted in a steady increase in both peak basal level and spiking rate in the presence of buffering, coupled with a steady increase in their corresponding σ values. A similar trend can be observed in the absence of buffering; however, after a threshold, the basal level continues to increase, while the spiking rate plateaus.
(TIF)

S1 Movie Video of network-wide astrocytic Ca^2+^ response to light stimulation. Video of the 10-by-10 network simulations performed in Fig 5 [Light stimulation: (◊) T = 4 s, δ = 45% (1.8 s); 4-by-4 central stimulated region is highlighted by the white box]. Simulations were conducted for 45 minutes in the presence (left panel) and absence (right panel) of Ca^2+^ buffering.
(MP4)
