# Supplementary figures and images for: Unraveling ChR2-driven stochastic Ca2+ dynamics in astrocytes: A call for new interventional paradigms
Source: PLoS Comput Biol. 2021 Feb 10;17(2):e1008648. doi: 10.1371/journal.pcbi.1008648 (PMC7875401; doi:10.1371/journal.pcbi.1008648)

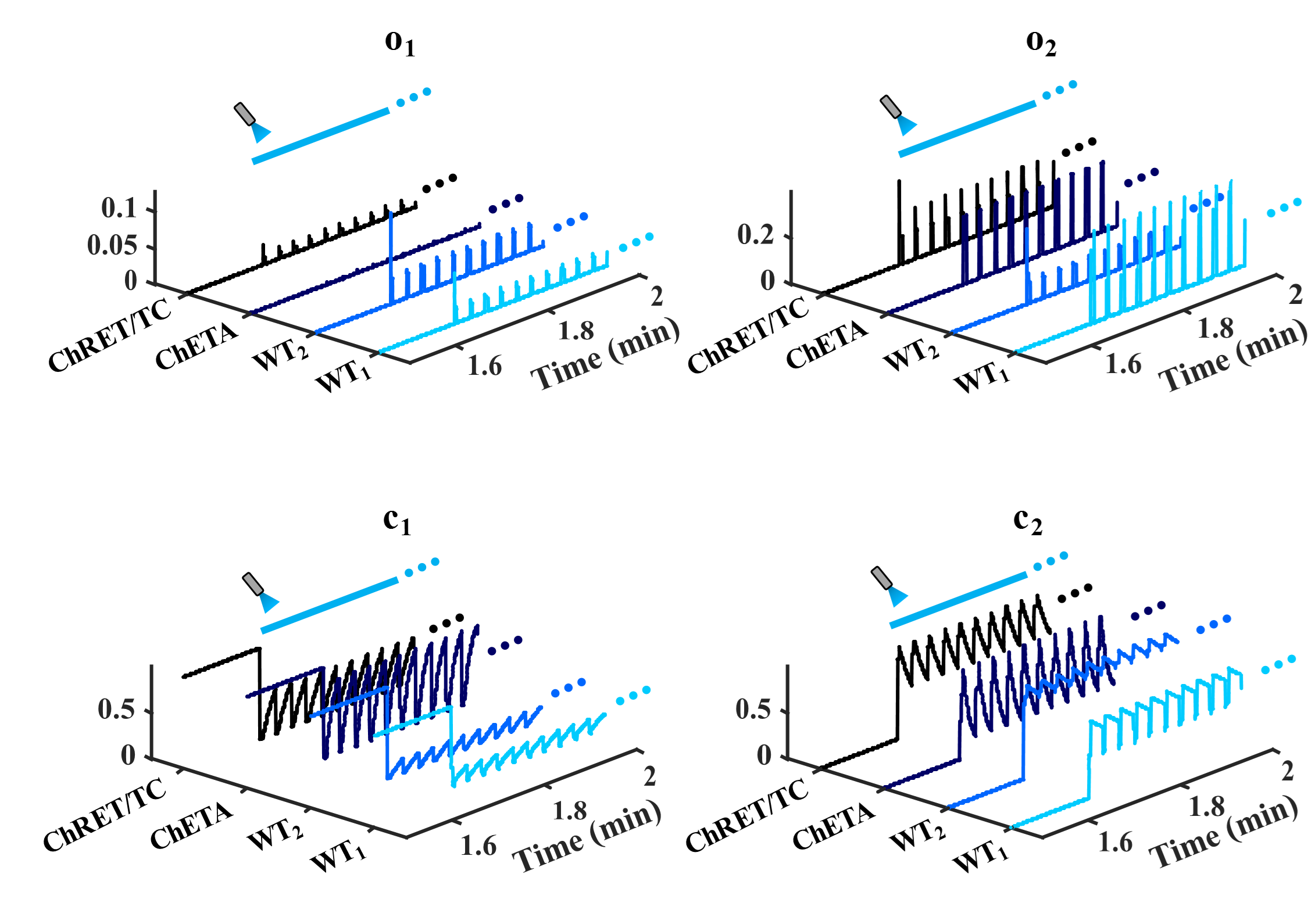

Supplement: S1 Fig — Representative traces of open state (o1 and o2) and closed state (c1 and c2) gating dynamics during light stimulation, corresponding to the 90-min simulation depicted in Fig 2 (wild type 1 (WT1), wild type 2 (WT2), ChETA. and ChRET/TC). A 30-second segment of each trace is shown to highlight details. Prior to light stimulation, all variants reside in the c1 state. Once stimulation is initiated, the variants reside in different states at varying levels. Within the open states, they mainly reside in o2. Solid horizontal blue line corresponds to the period during which the light stimulation was on. (TIF) [file pcbi.1008648.s001.tif]

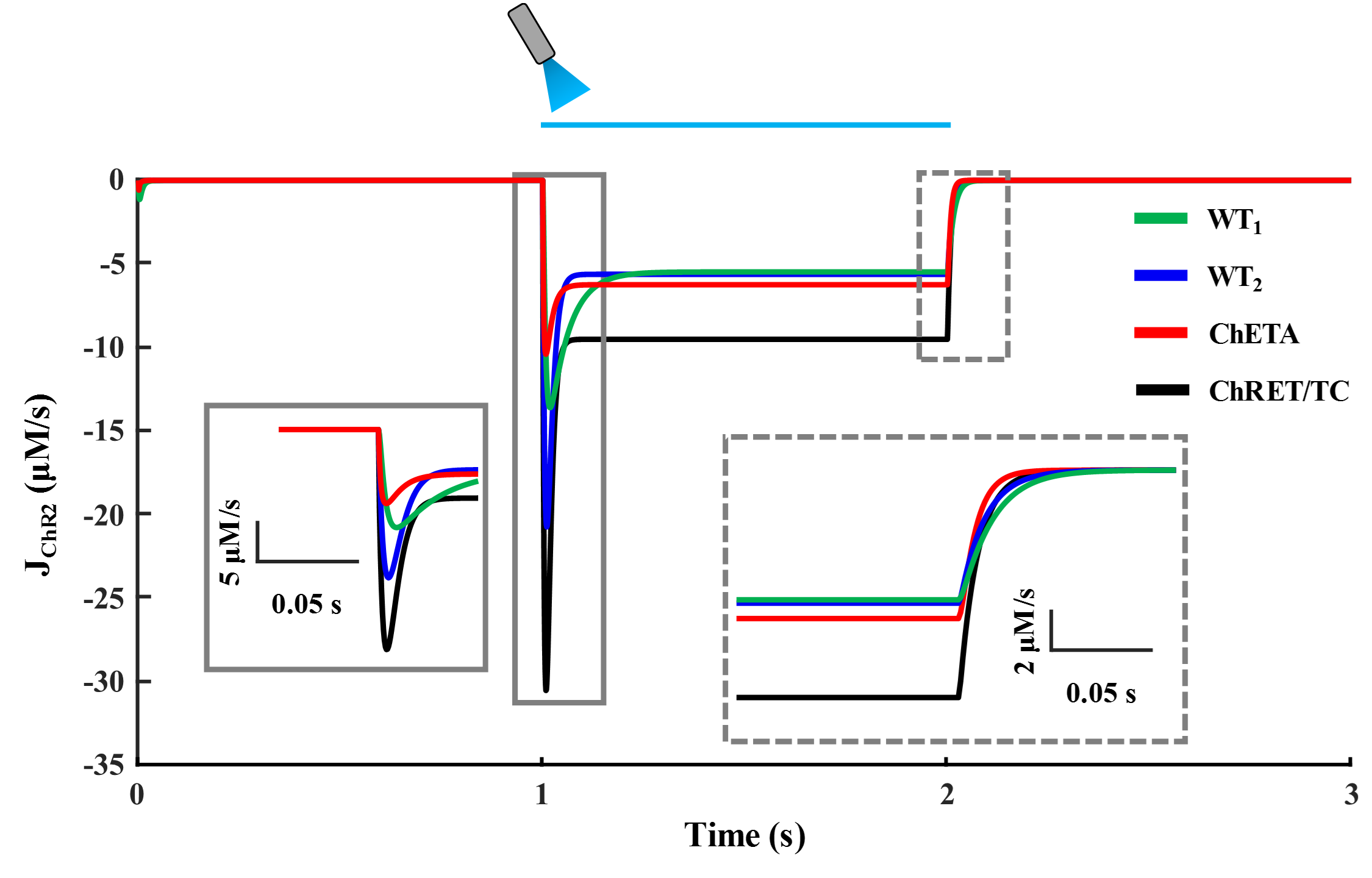

Supplement: S2 Fig — Simulated ChR2 channel flux (JChR2) in response to a 1-sec pulse stimulation. Solid horizontal blue line corresponds to the period during which the light pulse was on. The solid grey box highlights a transient phase, during which all variants exhibit a brief large-magnitude flux (ChRET/TC > WT2 > WT1 > ChETA), corresponding to the light-induced transition to the o1 state. The dashed grey box shows the plateau phase of the flux, corresponding to the transition and stabilization in the low-magnitude o2 state. The plateau phase flux magnitudes are in the order ChRET/TC > ChETA > WT2 > WT1. (TIF) [file pcbi.1008648.s002.tif]

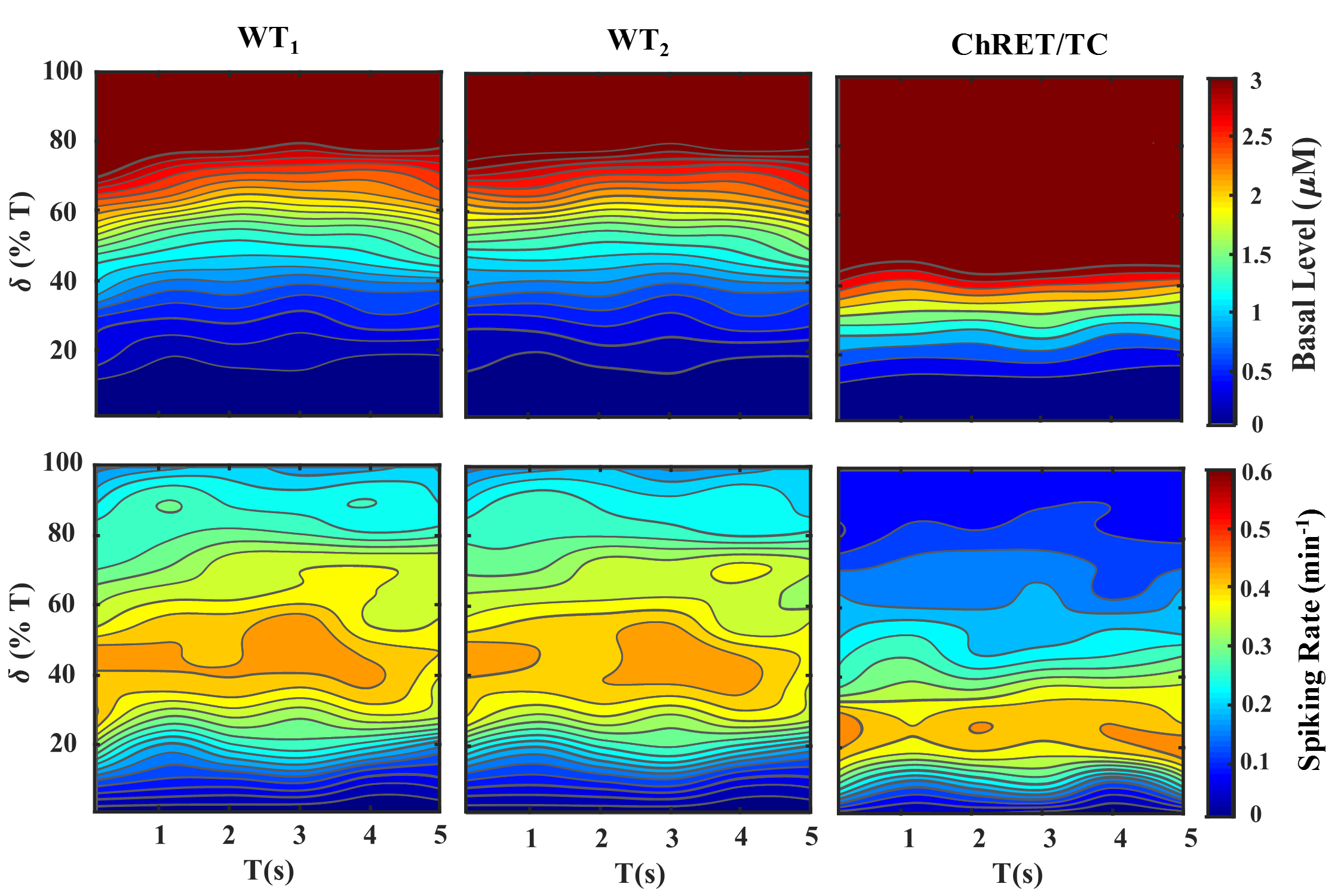

Supplement: S3 Fig — Simulations (45 min) were conducted to evaluate astrocytic Ca2+ response while expressing various ChR2 constructs. Different stimulation paradigms ranging from T = 1–5 s and δ = 0–100% of T (trials = 5) were applied from 100 seconds, until the end of the simulation. Each column corresponds to an evaluated ChR2 variant, i.e. WT1 (left), WT2 (center) and ChRET/TC (right). Heat maps of Ca2+ basal level (top panels) and spiking rate (bottom panels) for T-δ combinations are depicted. Scale bar for each heat map was capped to 3 μM and 0.6 spikes/min, respectively. For spike detection, a threshold of 0.2 μM above the basal level was utilized. Results show a similar basal level and spiking rate distribution for WT1 and WT2. However, ChRET/TC shows a smaller region of increased spiking activity coupled with a larger region of T-δ combinations eliciting supraphysiological basal level changes. (TIF) [file pcbi.1008648.s003.tif]

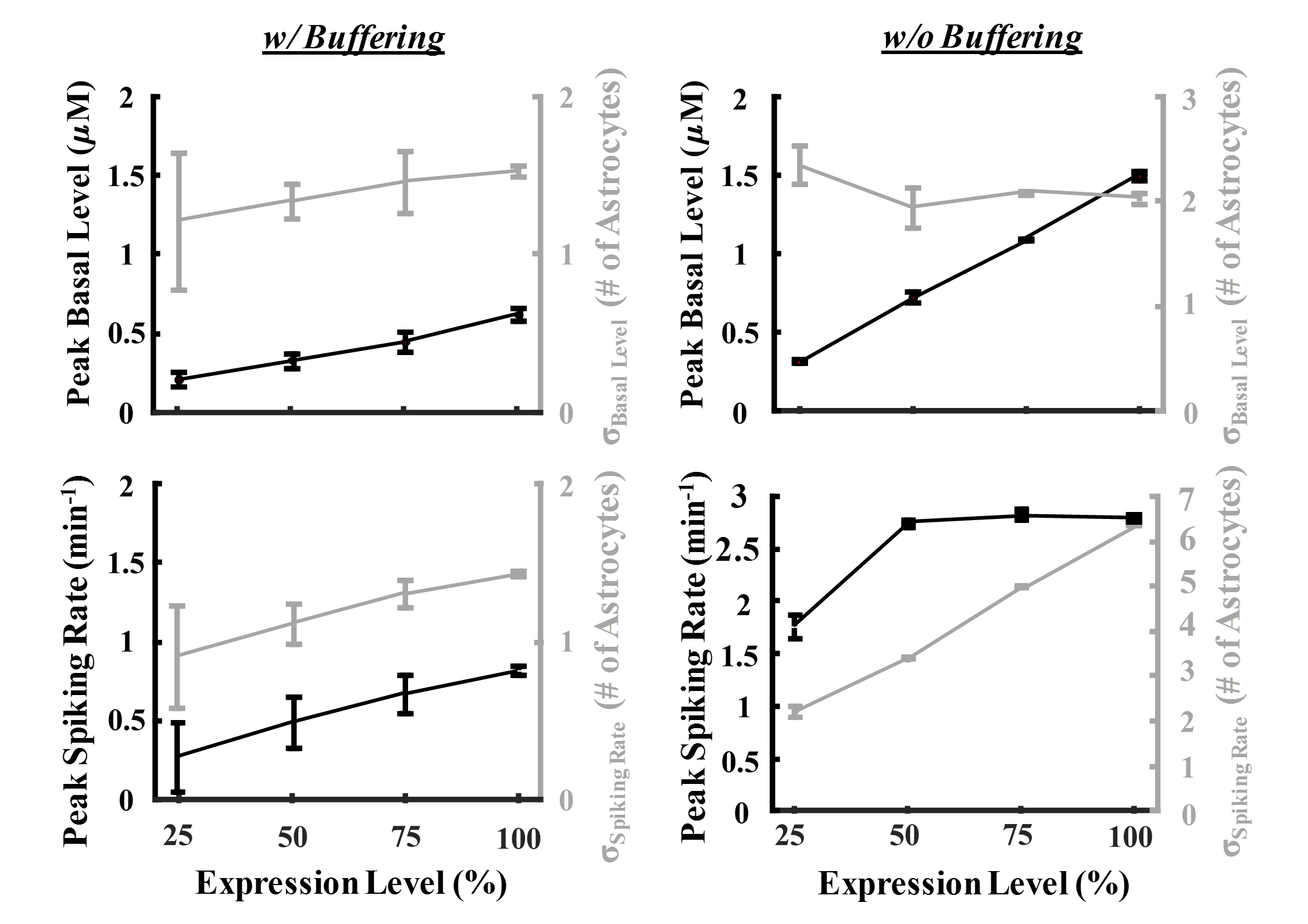

Supplement: S4 Fig — A 10-by-10 network of astrocytes was used to demonstrate the resulting response when the expression of the central 4-by-4 astrocytes (white square in Fig 5A and 5C) is varied [Light stimulation: (◊) T = 4 s, δ = 45% (1.8 s)]. Simulations were conducted for 45 minutes while the percentage of the central astrocytes randomly selected to express ChETA was varied (25, 50, 75, 100%) in the presence (left panel) and absence (right panel) of Ca2+ buffering. A total of 5 trials were conducted for each expression level. A symmetric 2D Gaussian fit was used to quantify the response, i.e. peak and magnitude of the spread from the stimulated region. Top row of plots shows the average and standard deviation of peak basal level and σbasal level as a function of expression. Bottom row of plots shows the average and standard deviation of peak spiking rate and σspiking rate as a function of expression. For spike classification, a threshold of 0.2 μM above basal level was selected. Increasing the number of cells expressing ChR2 in the stimulation region resulted in a steady increase in both peak basal level and spiking rate in the presence of buffering, coupled with a steady increase in their corresponding σ values. A similar trend can be observed in the absence of buffering; however, after a threshold, the basal level continues to increase, while the spiking rate plateaus. (TIF) [file pcbi.1008648.s004.tif]
